# The Mosaic Architecture of NRPS-PKS in the Arbuscular Mycorrhizal Fungus *Gigaspora margarita* Shows a Domain With Bacterial Signature

**DOI:** 10.3389/fmicb.2020.581313

**Published:** 2020-11-26

**Authors:** Francesco Venice, Alessandro Desirò, Gladstone Silva, Alessandra Salvioli, Paola Bonfante

**Affiliations:** ^1^Department of Life Sciences and Systems Biology, University of Turin, Turin, Italy; ^2^Institute for Sustainable Plant Protection (IPSP)-SS Turin–National Research Council (CNR), Turin, Italy; ^3^Department of Plant, Soil and Microbial Sciences, College of Agriculture and Natural Resources, Michigan State University, East Lansing, MI, United States; ^4^Department of Mycology, Federal University of Pernambuco, Recife, Brazil

**Keywords:** polyketides evolution, arbuscular mycorrhizal fungi, endobacteria, *Candidatus* Glomeribacter gigasporarum, Burkholderiaceae, NRPS-PKS, horizontal gene transfer

## Abstract

As obligate biotrophic symbionts, arbuscular mycorrhizal fungi (AMF) live in association with most land plants. Among them, *Gigaspora margarita* has been deeply investigated because of its peculiar features, i.e., the presence of an intracellular microbiota with endobacteria and viruses. The genome sequencing of this fungus revealed the presence of some hybrid non-ribosomal peptide synthases-polyketide synthases (NRPS-PKS) that have been rarely identified in AMF. The aim of this study is to describe the architecture of these NRPS-PKS sequences and to understand whether they are present in other fungal taxa related to *G. margarita*. A phylogenetic analysis shows that the ketoacyl synthase (KS) domain of one *G. margarita* NRPS-PKS clusters with prokaryotic sequences. Since horizontal gene transfer (HGT) has often been advocated as a relevant evolutionary mechanism for the spread of secondary metabolite genes, we hypothesized that a similar event could have interested the KS domain of the PKS module. The bacterial endosymbiont of *G. margarita*, *Candidatus* Glomeribacter gigasporarum (*Ca*Gg), was the first candidate as a donor, since it possesses a large biosynthetic cluster involving an NRPS-PKS. However, bioinformatics analyses do not confirm the hypothesis of a direct HGT from the endobacterium to the fungal host: indeed, endobacterial and fungal sequences show a different evolution and potentially different donors. Lastly, by amplifying a NRPS-PKS conserved fragment and mining the sequenced AMF genomes, we demonstrate that, irrespective of the presence of *Ca*Gg, *G. margarita*, and some other related Gigasporaceae possess such a sequence.

## Introduction

Fungi play crucial roles in the life on our planet: one of their most important and investigated feature is the production of secondary metabolites, which include polyketides, non-ribosomal peptides, terpenes, and indole alkaloids (Keller et al., 2005). Systematic studies on these products have led to the finding of an impressive number of useful bioactive molecules, like cyclosporins and statins, as well as potent poisons, like mycotoxins (Gallo et al., 2013).

Polyketide synthases (PKS) are mostly responsible for the production of polyketides. Their activity is finely regulated along the fungal life cycle by physiochemical environmental conditions as well as the competition with other microbes (Stroe et al., 2020). The genome sequencing of fungi from the Fungal Tree of Life^1^ has revealed that the genes encoding PKS are mostly arranged as biosynthetic gene clusters (BGCs). The latter are often associated with non-ribosomal peptide synthases (NRPS), which are also involved in siderophore formation (Carroll and Moore, 2018), leading to hybrid BGCs. These genomic regions are frequently co-regulated depending on the ecological function of their encoded product (Keller, 2019), meaning that their expression is modulated by environmental conditions related to a specific development stage of the fungus.

Non-ribosomal peptide synthases-polyketide synthases are produced by filamentous fungi mostly belonging to Dikarya, both to Pezizomycotina (Ascomycetes) and many Basidiomycetes. In addition to Fungi, an atlas of NRPS-PKS biosynthetic pathways enlarged the analysis to Bacteria and Archaea, examining a total of 2,699 genomes (Wang et al., 2014). Ascomycetes were confirmed to possess the highest number of BGCs among Fungi, while Bacteria showed the highest frequency of NRPS and PKS gene clusters when compared with Archaea or Eukarya. A phylogenomic analysis of 100 fungal genomes (Koczyk et al., 2015) showed that over 400 PKS originated from a burst of duplications in early Pezizomycotina, and also indicated potential horizontal transfers, pinpointing alternative donor–recipient scenarios. By contrast, information on early diverging fungi are more limited: a systematic review of publicly available non-Dikarya fungal proteomes (Sista Kameshwar and Qin, 2019) investigated the genome-wide annotations of 56 fungi belonging to *Glomeromycotina*, *Mucoromycotina*, *Mortierellomycotina*, *Zoopagomycota*, *Blastocladiomycota*, *Chytridiomycota*, *Neocallimastigomycota*, *Microsporidia*, and *Cryptomycota* from JGI-MycoCosm repository. This bioinformatic analysis reveals that the capacity to produce secondary metabolites is widespread also among the early diverging fungi. The results obtained from this comparative analysis show that arbuscular mycorrhizal fungi (AMF) (*Glomeromycotina*, according to Spatafora et al., 2016) exhibit a number of genes encoding for secondary metabolite biosynthesis, transport and catabolism.

Arbuscular mycorrhizal fungi are obligate biotrophs which associate with more than 72% of land plants (Brundrett and Tedersoo, 2018). While plant responses to fungal colonization have been deeply investigated, and many genetics and molecular bases underlying the mechanisms that control the establishment of the mycorrhizal symbiosis have been detected (Delaux et al., 2015; Lanfranco et al., 2018; Genre et al., 2020), the biological features of AMF have not been fully deciphered yet. The genome sequencing of some fungal species (Chen et al., 2018; Kobayashi et al., 2018; Morin et al., 2019; Sun et al., 2019; Venice et al., 2020) demonstrated that they possess limited capacity to degrade plant cell wall polymers, and they are auxotrophic for lipids and thiamine, since they lack fatty acid synthase and thiamine biosynthase. Indeed, as demonstrated in the AMF species *Rhizoglomus irregulare* (*sensu* Sieverding et al., 2014) (formerly classified as *Rhizophagus irregularis*), the treatment with a medium supported with myristate strongly pushes the growth and reproduction of these fungi so far described as unculturable (Sugiura et al., 2019). However, the secondary metabolites of *Glomeromycotina* have never been deeply investigated.

The AMF species *Gigaspora margarita* BEG34 has the largest fungal genome sequenced and annotated so far (more than 700 MB) (Venice et al., 2020). *G. margarita* BEG34 hosts a *Burkholderia*-related endobacterium (BRE) (Bonfante and Desirò, 2017), *Candidatus* Glomeribacter gigasporarum (*Ca*Gg) (Bianciotto et al., 2003), which contributes to shape some of the genetic features of the fungal host. Since NRPS-PKS sequences were found in the genome of *G. margarita*, here, we describe the architecture of one of them, and reveal that at least one of its domains is placed among prokaryotic sequences by phylogenetic analyses. Since secondary metabolite genes are often horizontally transferred (Koczyk et al., 2015), our first hypothesis involved a direct horizontal gene transfer (HGT) of the domain from the bacterial endosymbiont to *G. margarita*. Indeed, *Ca*Gg possesses a large biosynthetic cluster involving a NRPS-PKS. However, bioinformatic analyses did not confirm the direct HGT from the endobacterium to the fungus. Lastly, by amplifying a conserved fragment of the fungal NRPS-PKS from several Gigasporaceae isolates and mining the sequenced AMF genomes, we demonstrated that *G. margarita* and other related taxa possess such a sequence, regardless of the presence of the endobacterium. The results indicate that *G. margarita* genome has a chimeric mosaic structure where specific genes may have a bacterial signature, in addition and independent of the endobacterial presence.

## Results

As a first step of the investigation, the genome of *G. margarita* (Venice et al., 2020) was screened with the antiSMASH v.5 (Blin et al., 2019) and BIG-SCAPE (Navarro-Muñoz et al., 2020) pipelines for the identification of the three main enzyme classes that participate to the biosynthesis of secondary metabolites in fungi, that is PKS, NRPS, and NRPS-PKS hybrids (Keller, 2019). We found three Type 1 PKS (T1PKS) genes, nine NRPS-like genes and six hybrid NRPS/PKS, five of which consist of isolated genes. No similarities with known BGCs were observed for these fungal sequences.

### *Gigaspora margarita* PKS

**FIGURE 1:**
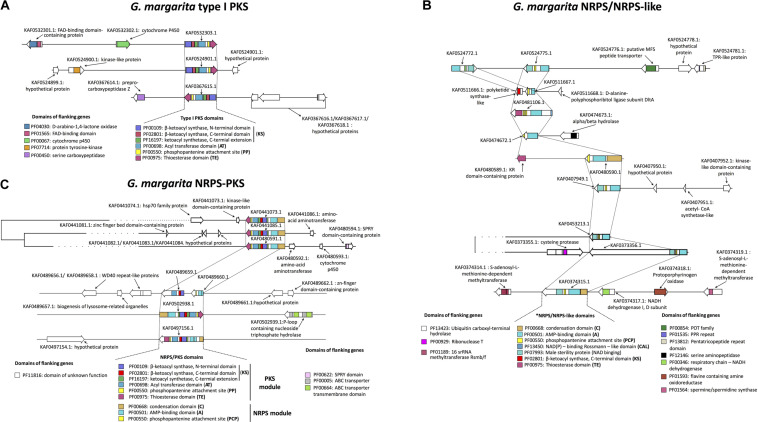
Predicted biosynthetic genes for secondary metabolites in *G. margarita*, including T1PKS **(A)**, NRPS/NRPS-like **(B)**, and hybrid NRPS-PKS **(C)**. A genomic window is shown for each gene according to the antiSMASH v.5 output (Blin et al., 2019), including flanking genes and their annotations (Venice et al., 2020). Core biosynthetic genes are connected by dashed lines, and a prediction of PFAM functional domains (shown in the legends) has been carried out with BIG-SCAPE (Navarro-Muñoz et al., 2020). Based on the BIG-SCAPE pipeline, some biosynthetic genes (AF0453213.1, KAF0373356.1, KAF0441073.1, KAF0441085.1, and KAF0480591.1) and their surroundings were clustered together due to >45% sequence similarity.

*Gigaspora margarita* possesses three T1PKS (Figure 1A). All the sequences have the same domain architecture. They all possess an Acyltransferase (AT) domain that incorporates the elongation group (i.e., malonyl-CoA, as predicted by antiSMASH) to an Acyl Carrier Protein (ACP). The ACP-bound group is then condensed by the β-ketoacyl synthase (KS) domain into the forming polyketide chain. The *G. margarita* sequences present a Phosphopantenine (PP) swinging tail that transfers the ACP-bound condensed product to the catalytic site, i.e., the C-terminal Thioesterase (TE) domain, which releases the final product and can influence its final structure (Newman et al., 2014). The identification of a putative final product, which is based on collinearity with highly characterized and publicly available sequences (Blin et al., 2019), did not return any result. However, due to the domain architecture, and to the absence of a domain that operates β-keto reduction, *G. margarita* PKS could be classified as Non-Reducing PKS (NR-PKS), which use an iterative mechanism to produce true polyketides. Fatty acid derivatives, which might be the alternative products, are instead produced by Partially and Highly reducing PKS (Cox, 2007); based on literature information, the starting substrate may be either a fatty acid, acyl-CoA, or another PKS products (Ray and Moore, 2016).

### *Gigaspora margarita* NRPS

The minimal composition of NRPS consists of an Adenylation (A) domain, a condensation (C) domain and a thioesterase or peptidyl carrier protein (T or PCP; Miller and Gulick, 2016). Through binding with adenosine monophosphate (AMP), the A domain selectively activates and incorporates amino acids into a growing product, tapping into a pool composed by the 20 proteinogenic amino acids, and up to 500 non-proteinogenic amino acids; the C domain is then responsible for the formation of peptide bonds (Walsh et al., 2013). The T or PCP domains release the final product, and possess a PP swinging tail that works as in PKS (see above). Only one *G. margarita* NRPS (KAF0480590.1) meets this canonical composition (Figure 1B). By contrast, the other eight sequences should be considered as NRPS-like, as they all lack a C domain. For example, KAF0374315.1 possesses the A and PCP domains, lacks a C domain, and is terminated by a reductase (NAD_binding_4). As demonstrated in *Trichoderma virens* (Mukherjee et al., 2012), the NAD_binding_4 domain converts a PCP-bound peptide to its corresponding primary alcohol. This domain is also similar to that of *Arabidopsis thaliana* MALE STERILITY 2 (MS2), which reduces palmitoyl-CoA to C16:0 alcohol, influencing exins development and determining pollen fertility (Wang et al., 2018). Almost all the other *G. margarita* NRPS-like contain the NAD_binding_4 domain, preceded by either A and PP (KAF0453213.1, KAF0407949.1, and KAF0373357.1) or A and ACP (KAF0524772.1 and KAF0524775.1) domains. The genomic context of two NRPS/NRPS-like genes (KAF0480590.1 and KAF0511667.1) suggests they may be part of ancestral, fragmented NRPS/PKS hybrid genes, as they are located in the immediate surroundings of genes with a predicted T domain (which is typical of PKS). However, these T domain-containing genes do not meet the minimal composition of PKS and it is unlikely that their assemblage with the NRPS-like genes results in a functional BGC. The same hypothesis could be formulated for KAF0481106.1, an NRPS-like gene that possesses a T domain itself, but does not meet the full composition of true NRPS/PKS hybrids.

In summary, the *G. margarita* NRPS-like products may be simple amino-alcohols, or alcohols of amino-acyl products (as for the case of KAF0524775.1). Finally, a *G. margarita* NRPS-like (KAF0481106.1) starts with an N-terminal Acyl-CoA ligase, which activates a carboxylic acid through binding with CoA. Based on the composition of the other domains in the same sequence, such a product could be transferred to an ACP and, finally, to a T domain containing a PP-binding region. Such organization is observed in several bacterial NRPS (Zhang et al., 2009), which, however, are larger and always contain a domain that allows the incorporation of the acyl-ACP product into a forming peptide, a feature that is missing in the *G. margarita* sequence.

### *Gigaspora margarita* NRPS-PKS

Non-ribosomal peptide synthases-polyketide synthases in *G. margarita* are more similar among them, compared to NRPS. They have comparable composition in terms of core domains, and have a higher degree of sequence similarity (Figure 1C). The NRPS module contains C and A domains, followed by a PP-binding site (KAF0502938.1) or a PCP domain (KAF0497156.1, KAF0480591.1, KAF0441072.1, and KAF0441085.1). The PKS modules in the same genes contain KS and AT domains, a PP-binding (KAF0502938.1) or a PCP domain (other), and an N-terminal T domain that releases the final product. The only exception is a putative NRPS-PKS BGC composed by KAF0489659.1 and KAF0489660.1, which results in an incomplete composition due to the lack of both C and T domains. The BIG-SCAPE analysis revealed that three hybrid NRPS/PKS genes (KAF0441073.1, KAF0441085.1, and KAF0480591.1) can be clustered together due to their sequence similarity and are thus likely to possess a phylogenetic relationship, such as paralogy.

We compared the PKS, NRPS and NRPS-PKS composition in AMF and related fungi, and found that no NRPS-PKS nor PKS are present in the genomes of other AMF besides *Gigaspora* (Table 1), whereas almost all possess NRPS or NRPS-like. Even if NRPS and NRPS-like seem to be ubiquitous in *Glomeromycotina*, we found limited similarities between *Gigaspora* and *Rhizoglomus* sequences. At least one NRPS gene belongs to a genomic region that is conserved in all sequenced *Rhizoglomus* species (Supplementary Figure S1), and that contains a sexuality-related HMG-box gene (Chen et al., 2018).

**TABLE 1:** PKS, NRPS/NRPS-like, and NRPS-PKS content in the sequenced genomes of Glomeromycotina and their relatives from Mucoromycota. The same screening was carried out for bacterial endosymbionts of Mucoromycota which genome is available. Reference studies for the analyzed genomic sequences are shown.

Organism	PKS	NRPS	NRPS-like	Hybrid NRPS-PKS*	References
*Gigaspora margarita* BEG34	3	1	8	6	Venice et al., 2020
*Gigaspora rosea* DAOM 194757	0	0	9	5	Morin et al., 2019
*Diversispora epigaea* IT104	0	0	2	0	Sun et al., 2019
*Rhizoglomus irregulare* DAOM 181602	0	1	1	0	Chen et al., 2018
*Rhizoglomus irregulare* A1	0	1	1	0	Chen et al., 2018
*Rhizoglomus irregulare* A4	0	1	0	0	Chen et al., 2018
*Rhizoglomus irregulare* C2	0	1	1	0	Chen et al., 2018
*Rhizoglomus irregulare* A5	0	1	0	0	Chen et al., 2018
*Rhizoglomus clarus* HR1	0	0	3	0	Kobayashi et al., 2018
*Glomus cerebriforme* DAOM 227022	0	1	1	0	Morin et al., 2019
*Rhizoglomus diaphanum* MUCL43196	0	1	1	0	Morin et al., 2019
*Mortierella elongata* AG-77	0	0	3	0	Uehling et al., 2017
*Jimgerdemannia lactiflua* OSC166217	0	0	3	0	Chang et al., 2019
*Jimgerdemannia flammicorona* GMNB39	0	0	1	0	Chang et al., 2019
*Jimgerdemannia flammicorona* AD002	0	0	2	0	Chang et al., 2019
*Endogone* sp. FLAS59071	0	0	2	0	Chang et al., 2019
*Rhizopus microsporus var. microsporus ATCC52813*	0	1	3	0	Mondo et al., 2017
*Candidatus* Glomeribacter gigasporarum (bacterial endosymbiont of *Gigaspora margarita* BEG34)	0	1	0	1	Ghignone et al., 2012
*Mycoavidus cysteinexigens* AG-77 (bacterial endosymbiont of *Mortierella elongata* AG-77)	0	3	0	0	Uehling et al., 2017
*Mycoavidus cysteinexigens* FMR23-6 I-B1 (bacterial endosymbiont of *Mortierella elongata* FMR23-6 I-B1)	0	0	0	0	Fujimura et al., 2014
MRE bacterial endosymbiont of *Rhizophagus clarus* NB112A	0	0	0	0	Naito et al., 2015
MRE bacterial endosymbiont of *Racocetra verrucosa* VA103A	0	0	0	0	Naito et al., 2015
MRE bacterial endosymbiont of *Claroideoglomus etunicatum* CA-OT135	0	0	0	0	Naito et al., 2015
*Mycetohabitans endofungorum* ATCC BAA-463	0	3	0	0	Johnson et al., 2015
*Burkholderia xenovorans* BXA		2			Chain et al., 2006
*Paraburkholderia rhizoxinica* HKI 454	4	16	0	2	Lackner et al., 2011
*Burkholderia phymatum* STM815	0	3	0	1	Moulin et al., 2014

### *Gigaspora margarita* Putative BGCs

Genes for the biosynthesis of secondary metabolites may be present as isolated genes or in tandems, which may be referred to as BGCs and are often co-regulated and participate to the concerted biosynthesis of a single product (Keller, 2019).

Polyketide synthases, NRPS, and NRPS-PKS in *G. margarita* mostly consist of isolated genes, with two exceptions: KAF0524772.1 and KAF0524775.1 are two NRPS genes that form a putative BGC in the same 45 Kbp genomic region, while one NRPS-PKS consists of two neighboring genes within a window of about 47 Kbp (KAF0489659.1 and KAF0489660.1).

We wondered whether such genes showed a co-regulation, as is often true for BGC (Keller, 2019). We performed a co-regulation analysis on a set of 24 RNA-seq libraries from different stages of *G. margarita* life cycle (Supplementary Table S1). The algorithm divided the 26,604 *G. margarita* genes in 4,950 virtual groups based on their co-expression values, which ranged from 0 (no correlation) to 1 (full correlation). No co-expression was observed for either of the four genes belonging to putative BGCs. By contrast, one group of co-expressed genes among those with better support (i.e., average correlation >0.6), contained two PKS genes that are located on different genomic scaffolds (KAF0532303.1 and KAF0524901.1). These genes were among the top 15 co-expressed genes present in the group (Supplementary Figure S2A), which contained a total of 568 genes. The list included a cytochrome P450 (KAF0532302.1) located directly upstream of KAF0532303.1. Given the known role of cytochrome P450 in fungal and bacterial secondary metabolites biosynthesis (Chadha et al., 2018; Shin et al., 2018), this could be an evidence of enzymatic cooperation between KAF0532302.1 and the two PKS. Other genes consistently co-regulated with the two PKS included a dihydroxy-acid dehydratase which may participate in CoA production, a general substrate transporter, and a deacetylase with chitin or peptidoglycan as predicted substrates.

Among the NRPS and NRPS-PKS genes, only one was found in a group meeting an average correlation >0.6. This group gathered 430 genes, and the NRPS-PKS gene was found among the top 40 co-regulated genes (KAF0441072.1; Supplementary Figure S2B). As described for KAF0532302.1 and KAF0532303.1, a cytochrome P450 and a general substrate transporter are among the co-regulated genes, together with an alpha/beta hydrolase. However, no co-regulation with other secondary metabolites-producing genes was observed.

In summary, mining the genome of *G. margarita* led to the discovery of three PKS, nine NRPS/NRPS-like, five NRPS-PKS hybrids, and three situations where NRPS-like and PKS-like genes co-localize, but lead to a likely incomplete BGC due to the absence of key domains. The data confirmed the analysis by Sista Kameshwar and Qin (2019), claiming that most Mucoromycota genomes encode for NRPS and NRPS-like genes, while PKS are less common and present in lower numbers. However, the only species that seems to encode for hybrid NRPS-PKS is *Gigaspora rosea*, the closest sequenced relative of *G. margarita*. This result has also been confirmed through a genome-scale phylogeny, including all the sequenced Glomeromycotina, that showed that NRPS-PKS belong to a recently expanded gene family exclusive of the two so far sequenced *Gigaspora* species (Venice et al., 2020).

### NRPS-PKS in the Endobacterium of *Gigaspora margarita*

*Gigaspora margarita* BEG34 harbors a population of obligate and vertically transmitted endobacteria named *Ca*Gg (Bianciotto et al., 2003). As other obligate endosymbionts, *Ca*Gg possesses a reduced genome, which couples with a nutritional dependence on its fungal host (Ghignone et al., 2012).

**FIGURE 2:**
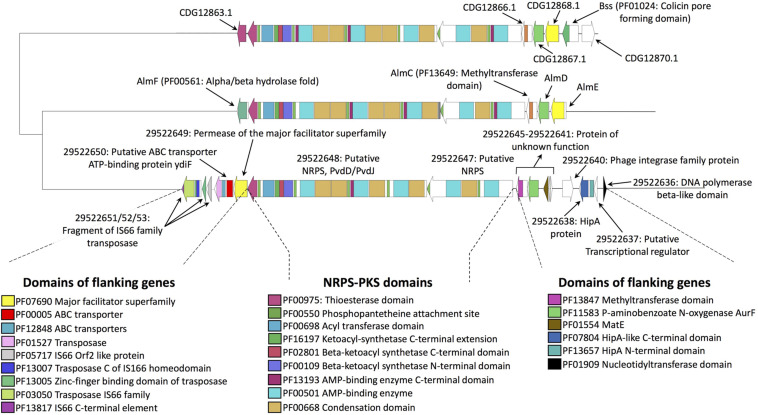
Genomic region containing the putative biosynthetic cluster in the *Ca*Gg endobacterium. The cluster contains a NRPS-PKS gene and a neighboring NRPS (29522648 and 29522647, respectively). Protein IDs were retrieved from the MicroScope MaGe platform (https://mage.genoscope.cns.fr/microscope/ home/index.php). A similarity was found with the MIBiG reference clusters for the biosynthesis of althiomycin of *Serratia marcescens* and *Myxococcus xanthus* (BGC0000955.1 and BGC0001415.1, respectively). The core biosynthetic genes are linked with dashed lines. Sequence alignment, as well as the distance tree and the PFAM domains prediction, were obtained with BIG-SCAPE.

To understand whether NRPS-PKS sequences of the fungus are shared with its endobacterium, an antiSMASH analysis was performed on the bacterial genome. The analysis detected the presence of a large gene cluster (around 65 Kbp; Figure 2) composed by an NRPS (protein ID: 29522647) and a NRPS-PKS (protein ID: 29522647). The NRPS possesses two domains: one with an A and PCP domains, and glycine as putative substrate, and the second with a C, A, and PCP domains, with cysteine as the putative substrate. The NRPS portion of 29522648 consists of three domains: two have a C-A-PCP organization, which is followed by an Epimerization (E) domain in the other domain. The predicted substrates are serine and D-cysteine. Notwithstanding a similar domain composition, the *Ca*Gg cluster shows limited sequence similarity with any of the *G. margarita* sequences, as already evident due to its size and the lack of BLAST homology (Supplementary Table S2). By contrast, it has homology with the MIBiG BGC0001415.1 and BGC0000955.1 reference clusters for the biosynthesis of althiomycin, an antibiotic produced by *Serratia marcescens* and the bacterial predator *Myxococcus xanthus* (Cortina et al., 2011; Gerc et al., 2012). A transposon (protein ID: 29522626) is also present in the genomic region surrounding the *Ca*Gg gene cluster; as already observed, transposons may mediate the HGT of even large gene clusters from distantly related bacteria (Hagen et al., 2018).

Hybrid NRPS-PKS sequences have not been found in the genome of *Mycoavidus cysteinexigens*, a bacterial endosymbiont related to *Ca*Gg (Ohshima et al., 2016; Uehling et al., 2017), nor in its fungal host *Mortierella elongata* (Uehling et al., 2017), a taxon of Mortierellomycotina (Mucoromycota) closely related to AMF (Spatafora et al., 2016). By contrast, *Paraburkholderia rhizoxinica*, the endobacterium of *Rhizopus microsporus* (Mucoromycotina), encodes for two NRPS-PKS that are involved in the biosynthesis of the phytotoxin rhizoxin (Lackner et al., 2011). The genomes of the closest *Ca*Gg relatives with free-living capabilities, *Mycetohabitans endofungorum* and *Burkholderia xenovorans* (Table 1), do not encode for hybrid NRPS-PKS. An exception is represented by *Burkholderia phymatum*, a nitrogen-fixing bacterium (Moulin et al., 2014) that codes for a NRPS-PKS. As expected, no secondary metabolites genes have been found in the strongly reduced genomes of the *Candidatus* Moeniiplasma glomeromycotorum (Naito et al., 2017), a different taxon of bacterial endosymbiont hosted in AMF and other Mucoromycota lineages.

The genome of the *Ca*Gg endobacterium of *G. margarita* contains a BGC that, based on its size and on BLAST results, does not seem to be the related to those of its fungal host. The highest similarity for this BGC is found in bacterial groups that are distant from *Ca*Gg.

### The Sequence of *G. margarita* NRPS-PKS Reveals Homologies With Sequences From Free-Living Bacteria

Horizontal gene transfer events have been hypothesized to be important for secondary metabolite production in fungi (Koczyk et al., 2015), and potential HGT events have been identified in the genome of *G. margarita* (Venice et al., 2020). Thus, we wondered about the prokaryotic or eukaryotic origin of *G. margarita* NRPS-PKS sequences.

Due to its high expression level in all the fungal life stages (Venice et al., 2020), KAF0502938.1 from *G. margarita* was selected to perform a BLASTp (Supplementary Table S2). The search retrieved almost exclusively bacterial sequences, while no sequences from Mucoromycota (besides *Gigaspora*) or Dikarya were present in the results. The top BLAST hits were the ones with the chitin-degrading bacterium *Archangium gephyra* (Sharma and Subramanian, 2017), but the list of potential homologs also included proteins from nitrogen-fixing bacteria, as well as from a few *Bacillus* and *Pedobacter* species. Betaproteobacteria were under-represented, and this is contrary to the evidence that these bacteria are very common endosymbionts in Mucoromycota including Glomeromycotina (Bonfante and Venice, 2020).

As a whole, the analysis excluded relevant similarities of *G. margarita* sequences with those of other early diverging or Dikarya fungi, while revealing a relatedness with sequences belonging to free-living bacteria.

### HGT Inference Through Phylogenetic Reconstructions

Since the previous analyses revealed that, among AMF, NRPS-PKS seem to be limited to the genus *Gigaspora*, and the *G. margarita* KAF0502938.1 has homology with bacterial sequences, we further investigated the putative HGT origin of such NRPS-PKS sequences in *G. margarita* and *G. rosea*. We used a phylogenetic approach involving both fungal and bacterial sequences. A phylogeny was built starting from the NRPS-PKS of both *G. margarita* and its *Ca*Gg endobacterium (KAF0502938.1 and 29522648, respectively), together with the homolog from *G. rosea* (RIB14068.1). Since NRPS-PKS are highly modular and variable in the composition of their domains, we reconstructed the phylogenetic models based on single domains, rather than full length sequences. We choose the KS and A domains as representatives of the PKS and NRPS modules, respectively. Since the KS domain is present in both PKS and NRPS-PKS, and the sequence selection was based on BLAST homology, several PKS were also included in the tree along NRPS-PKS.

**FIGURE 3:**
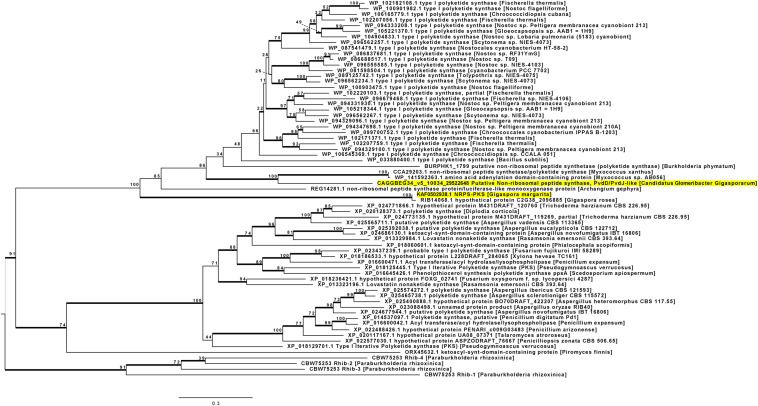
ML tree based on the KS domain of KAF0502938.1, a hybrid NRPS-PKS of *G. margarita*. The sequence of *G. margarita*, as well as that of its endobacterium, *Ca*Gg, are highlighted. The sequences of *G. margarita* and *G. rosea* are gathered into a bacterial clade which is well separated from the Dikarya group. The sequence of the *Ca*Gg endobacterium clusters into a sub-clade which is well separated from the *Gigaspora* sequences, but which contains sequences from *Myxococcus*. Despite its homology with the *Gigaspora* sequences, the PKS from the basal fungus *P. finnis* seems to have a fungal signature. Bootstrap support values are shown on the tree nodes. Thick lines indicate Bayesian posterior probability ≥95%.

Sixty-seven sequences were included in the phylogenetic reconstruction. With the exception of *Gigaspora*, KAF0502938.1 does not have any fungal sequence among the best BLAST hits (Supplementary Table S2). To further confirm that the origin of the *Gigaspora* sequence is outside of the fungal lineage, we included distant fungal homologs, by using a taxonomy-oriented BLAST search. In addition, sequences of *Burkholderia*-related bacteria were introduced in the set, despite their low BLAST homology with KAF0502938.1. This bacterial group has in fact a well-known history of co-existence with Mucoromycota (Bonfante et al., 2019), and the *Ca*Gg endobacterium is *Burkholderia*-related. Therefore, these sequences were used to test whether the evolution of NRPS-PKS retraces such symbiotic history. The resulting tree is shown in Figure 3.

The automated procedure of sequence selection (see section “Materials and Methods”) confirmed the absence of PKS or hybrid NRPS-PKS in all Mucoromycota besides *Gigaspora*, with the exception of *Pyromyces finnis*. The Maximum Likelihood (ML) and Bayesian phylogenetic analyses highlighted a separation between KS domains of other fungi, and those belonging to bacteria and *Gigaspora* species together. The only other Mucoromycota fungus included in our selection, *P. finnis*, is an anaerobic fungus from Neocallimastigales (Haitjema et al., 2014); its KS domain, however, seems to possess a fungal signature.

The *Gigaspora* KS domains form a separate clade with no affiliation with either bacterial or fungal sequences, indicating that none of the BLAST hits found in Supplementary Table S2 are to be considered donor sequences, including the best BLAST hit from *A. gephyra*, REG14281.1. By contrast, the sequence of *Ca*Gg has well supported phylogenetic relatedness with the sequences from two *Myxococcus* species, which confirms their similarities in terms of domains composition (see section “NRPS-PKS in the Endobacterium of *Gigaspora margarita*”). *A. gephyra* belongs to myxobacteria as well, but its placing in the phylogenetic tree suggests that its sequence has diverged earlier than those of *Ca*Gg and *Myxococcus*. The placement of the KS domain of *B. phymatum*, and of the rhizoxin-related sequences from *P. rhizoxinica*, is poorly supported or external to the main clades, indicating unrelatedness with the included sequences. According to the reconstruction in Figure 3, twenty-nine highly diversified sequences from Cyanobacteria may share the same common ancestor of the *Ca*Gg-*Myxococcus*-*B. phymatum* group. Secondary metabolites genes in Cyanobacteria have already been described as spectacularly diversified and frequently involved in HGT events (Calteau et al., 2014), but their similarities with members of Glomeromycota and their associated endobacteria need further investigation.

To further validate our phylogenetic reconstruction, we generated three additional ML trees (Supplementary File S1) with different constraints: in the first reconstruction, the two *Gigaspora* and all bacteria were forced to be monophyletic; in the second analysis, we assumed a monophyly between all fungi (i.e., *Gigaspora*, Dikarya, and *P. finnis*). The first constraint was used to enforce the hypothesis of HGT from bacteria to Gigaspora, while the second was used as null hypothesis. All the models were tested with several statistical tests including the Approximately Unbiased test, a standard procedure in validating HGT events for metabolic gene clusters in fungi (Wisecaver and Rokas, 2015). The unconstrained phylogeny, together with the first constrained model, i.e., the one assuming monophyly between *Gigaspora* and bacteria, had the best log-likelihood scores and was accepted by all tests, while the null hypothesis was rejected (Supplementary File S1).

The same procedure of sequence selection was used to build up a dataset based on the A domain of the KAF0502938.1 NRPS module, but low support values were obtained (Supplementary Figure S3). NRPS are more widespread than PKS in Mucoromycota and their associated bacteria (Table 1); for this reason, we were able to include a higher number of sequences from close relatives of *Ca*Gg, such as *M. cysteinexigens* and *M. endofungorum*, while no significant similarities were detected between the *Gigaspora* A domains and those of other Mucoromycota. Both Bayesian and ML reconstructions indicated no relatedness between the *Gigaspora* domains and bacterial domains, highlighting a fungal signature. By contrast, the placement of *Ca*Gg had high support values along the tree: its A domain appeared to be unrelated to those of its close relatives, but again clustered with the same *Myxococcus* genes included in the KS-based tree (Figure 3).

In conclusion, the analysis suggested a potential HGT-mediated hybridization of secondary metabolites genes in *Gigaspora*, as the two phylogenies built for different domains of the same sequences gave different results. The HGT event observed for the KS domain, however, seem to be independent of the *Ca*Gg endobacterium and related bacteria, which are unlikely to be the potential donors. Sequences from *Myxococcus* have strong relatedness with *Ca*Gg, but not with *G. margarita*, supporting the hypothesis of separate HGT events.

### Homologs of *G. margarita* NRPS-PKS Are Present in the Genome of Several Gigasporaceae Taxa

**FIGURE 4:**
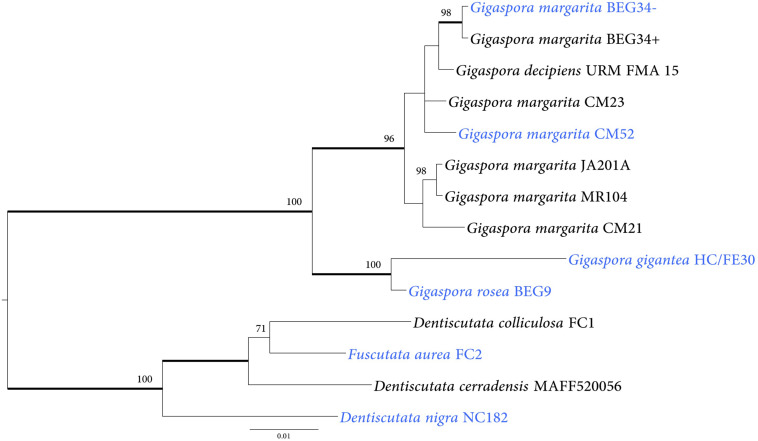
Phylogenetic placement of NRPS-PKS sequences identified in 14 AMF isolates. NRPS-PKS sequences cluster into two main clades. The first clade encompasses sequences from species in the Gigasporaceae family; the second clade includes sequences from taxa that belong to the Dentiscutataceae family. The tree shows the topology obtained with the Bayesian method; branches with Bayesian posterior probabilities ≥0.95 are thickened and ML bootstrap support values ≥70 are shown. The isolates shown in blue color do not host *Ca*Gg. *Gigaspora margarita* BEG34- and *G. margarita* BEG34+ are isogenic, but the first was artificially cured from its endobacterium (Lumini et al., 2007).

We investigated 28 AMF isolates that belonged to several genera of Gigasporaceae (Table 2) in order to understand if the HGT event occurred in other fungal taxa related to *G. margarita* BEG34. Among them, eighteen isolates hosted *Ca*Gg whereas ten were devoid of it (Table 2). PCR amplification was performed by selecting a 732 bp fragment from the hybrid NRPS-PKS KAF0502938.1. The fragment was successfully amplified from 14 fungal isolates, nine of which were associated with *Ca*Gg. Phylogenetic reconstructions (Figure 4) generated a tree that mirrored the Gigasporaceae phylogeny (Supplementary Figure S4). Indeed, taxa within Dentiscutataceaeae clustered together and were sister to the Gigasporaceae clade that encompassed all *Gigaspora* spp. isolates. These findings showed that the presence of the NRPS-PKS gene is not a unique feature of *G. margarita* BEG34, but it is shared by different taxa in Gigasporaceae.

**TABLE 2:** List of isolates used in the PCR screening for NRPS-PKS in Gigasporales.

Species	Isolate/Voucher	Origin	CaGg presence	References
*Cetraspora helvetica*	SAF15	Switzerland	−	this study
*Cetraspora pellucida*	MAFF520083	Japan	−	this study
*Cetraspora pellucida*	MN408A	United States	✓	Mondo et al., 2012
*Cetraspora pellucida*	BR208A	Brazil	✓	Mondo et al., 2012
*Cetraspora pellucida*	CL750A	Colombia	✓	Mondo et al., 2012
*Dentiscutata cerradensis*	MAFF520056	Japan	✓	this study
*Dentiscutata colliculosa*	FC1*	Brazil	✓	this study
*Dentiscutata nigra*	NC182	United States	−	this study
*Fuscutata aurea*	FC2*	Brazil	−	this study
*Fuscutata heterogama*	FC3*	Brazil	✓	this study
*Fuscutata heterogama*	URM FMA 06	Brazil	−	this study
*Gigaspora decipiens*	URM FMA 15	Brazil	✓	this study
*Gigaspora gigantea*	HC/FE30	United States	−	Bianciotto et al., 2000
*Gigaspora margarita*	CM21	Cameroon	✓	Desirò et al., 2014
*Gigaspora margarita*	CM23	Cameroon	✓	Desirò et al., 2014
*Gigaspora margarita*	CM52	Cameroon	✓	Desirò et al., 2014
*Gigaspora margarita*	JA201A	Japan	✓	Mondo et al., 2012
*Gigaspora margarita*	MR104	Morocco	✓	Mondo et al., 2012
*Gigaspora margarita*	BEG34+	New Zealand	✓	Bianciotto et al., 2000
*Gigaspora margarita*	BEG34−	New Zealand	−	Lumini et al., 2007
*Gigaspora rosea*	BEG9	United States	−	Bianciotto et al., 2000
*Racocetra castanea*	BEG1	France	✓	Mondo et al., 2012
*Racocetra coralloidea*	CA260	United States	✓	this study
*Racocetra fulgida*	FC6*	Brazil	✓	this study
*Racocetra fulgida*	IN212	United States	✓	this study
*Racocetra gregaria*	NC210	United States	−	this study
*Racocetra verrucosa*	HA150A	United States	✓	Mondo et al., 2012
*Scutellospora calospora*	AU212A	Australia	−	this study

Species, isolate/voucher, place of origin, CaGg presence, and reference study reporting the presence/absence of CaGg are shown.

*Field collected isolate.

## Discussion

The genome mining of *G. margarita* has revealed the presence of genes involved in the biosynthesis of secondary metabolites, a class of compounds that have a crucial role in pathogenic fungi (Keller, 2019), but have been poorly investigated in symbiotic ones. Indeed, PKS and their biosynthetic genes have been identified in lichenizing, ectomycorrhizal, and ericoid fungi (Bertrand and Sorensen, 2018; Martino et al., 2018; Armaleo et al., 2019), whereas the molecular determinants leading to such biosynthetic activity have been rarely considered in studies of AMF genomes (Venice et al., 2020).

By using *in silico* analyses, we demonstrate that PKS, NRPS, and NRPS-PKS sequences are present in *G. margarita* genome and, at least in the case of two PKS (KAF0532303.1 and KAF0524901.1), they show patterns of co-expression along the fungal life cycle, suggesting they act as a BGC. This gene equipment is also similarly present in the genome of *G. rosea* (Morin et al., 2019), the most closely related AMF taxon to *G. margarita*.

Looking at the genome scale phylogeny of AMF as reconstructed by orthologous-based data (Sun et al., 2019; Venice et al., 2020), the NRPS-PKS result to be absent in the genomes of the widespread Glomerales, and present in Gigasporaceae. The reason for their absence in Glomerales genomes might be explained by an ancient loss of these fungal sequences. The alternative scenario could be represented by an HGT event that only involved the common ancestor of Gigasporaceae and Dentiscutataceaeae as recipient, without entailing Glomerales and other AMF taxa. This could be related to the peculiar and still poorly understood dynamics that make *Gigaspora* genomes weakly shielded against the insertion of foreign DNA, as demonstrated by their expanded genomes that are dominated by transposons (Morin et al., 2019; Venice et al., 2020). In this context, Gigasporaceae could act as recipient from many potential donors: other soil fungi, associated free-living bacteria, as well as their interacting-host plant. As other AMF, Gigasporaceae are in fact component of the plant microbiota, but, in the meantime, they also host their own microbiota (Bonfante et al., 2019). In addition to the two classes of endobacteria thriving in Gigasporaceae (Desirò et al., 2014), AMF are colonized by many saprotrophic bacteria that live at the spores and hyphal surface, as seen under transmission electron microscope (Bonfante and Anca, 2009), and identified in some AMF isolates (Naumann et al., 2010; Agnolucci et al., 2019).

On the basis of these considerations, we hypothesized that other not yet sequenced Gigasporaceae and Dentiscutataceaeae may contain such NRPS-PKS genes. Experimental results confirmed the hypothesis, as a successful amplification of a fragment located in the PKS module of a NRPS-PKS gene has been achieved from 14 out of 28 Gigasporaceae isolates analyzed in this study. Furthermore, the phylogenies obtained with the NRPS-PKS and common AMF markers showed similar topologies, suggesting that the gene acquisition might have occurred before the diversification of taxa within the genera *Dentiscutata* and *Gigaspora*.

A detailed investigation of the *G. margarita* KAF0502938.1 NRPS-PKS sequence revealed a complex mosaic structure. While the A domain from the NRPS module clustered with other fungal sequences, the KS domain from the PKS module of the same sequence showed a clear prokaryotic signature. Our first and simplest hypothesis was that the bacterial endosymbiont of *G. margarita* had transferred this domain to its fungal host through HGT events. Indeed, we demonstrated that *Ca*Gg possesses NRPS-PKS sequences coding for a hypothetical antibiotic-like compound. However, different *in silico* tools did not confirm a direct HGT from *Ca*Gg as a donor and *G. margarita* as a recipient. While the prokaryotic origin of the fungal domain remains unsolved, *Ca*Gg NRPS-PKS can be considered as a close relative of *Myxococcus* sequences. Indeed, phylogenetic reconstructions for both A and KS domains point to the same relatedness. *M. xanthus*, the model species for Myxobacteria, is a predatory bacterium that feeds on other bacteria and even fungi (Thiery and Kaimer, 2020), a capacity which is mediated by induced prey lysis from the outside. Such a trophic behavior may facilitate HGT events through the integration of undegenerated prey DNA (Goldman et al., 2006): this leaves opens the question about alternative donor and recipient scenarios for *Ca*Gg and *M. xanthus* in our reconstruction. In addition, the exchange of the bacterial NRPS-PKS sequences might have been supported by the presence of a transposon located in the proximity of the *Ca*Gg gene, acting as a vehicle for HGT, as reported in other bacterial models (Hagen et al., 2018).

In conclusion, according to the view that eukaryotic genomes are evolutionary chimeras with most of the genes stemming from bacteria (Brueckner and Martin, 2020), *G. margarita* appears to be a complex organism where nuclear and mitochondrial fungal sequences co-exist with viral and endobacterial ones: all these features give rise to a genome identified as a metagenome (Venice et al., 2020). The detailed analysis of a class of genes encoding for enzymes leading to polyketides, non-ribosomal peptides, and hybrid NRPS-PKS showed a further level of complexity. Indeed, NRPS-PKS sequences, which are in themselves hybrid sequences, contain modules with prokaryotic signatures, confirming the mosaic structure of this fungal genome. It seems that *G. margarita* and *Ca*Gg genomes have been built up by a number of molecular pieces, which - as these NRPS-PKS sequences - likely originated from separate evolutionary events.

## Materials and Methods

### Prediction of Secondary Metabolites Genes in the *G. margarita* and *Ca*Gg Genomes

The identification of *G. margarita* genes involved in secondary metabolites production was performed with antiSMASH v.5 (Blin et al., 2019). The parameters used were “–cb-general –cb-known clusters –cb-subclusters –asf –pfam2go –smcog-trees –taxon fungi” and the inputs were the *G. margarita* assembled scaffolds and gene annotations in GFF3 format, as retrieved from the NCBI BioProject PRJNA575165. The output of antiSMASH was then fed into the BIG-SCAPE (Navarro-Muñoz et al., 2020) to annotate the functional domains of the candidate genes and to verify their co-relatedness, and relatedness with known BGCs present in the MIBiG repository. The chosen similarity threshold for clustering of related genes or BGC was 50%. The same procedure was applied for the *Ca*Gg genome, found at https://mage.genoscope.cns.fr/microscope/home/index.php, and for all the genomes presented in Table 1.

### Genes Co-expression Analysis

The co-expression analysis has been performed in the R environment with the WGCNA package (Langfelder and Horvath, 2008). The absolute read counts-per-gene were obtained with salmon v.0.13.1 (Patro et al., 2017) as described in Venice et al., 2020. Briefly, the libraries (Supplementary Table S1) were obtained from different fungal life stages, both pre-symbiotic (spores germinating in presence or absence of GR24, a synthetic analog of strigolactones, used to simulate plant signals) and symbiotic (intra- and extra-radical mycelium from mycorrhizal roots of *Lotus japonicus*). The R^2^ cutoff was set to 0.9 in order to choose the soft thresholding power needed for adjacency calculation and topological overlap matrix (TOM) construction. Among the predicted co-expressed gene groups, those showing a correlation of >60% were selected. For each of the two co-expressed gene groups shown in Supplementary Figures S1, S2, only 15 and 40 top co-expressed genes were selected. The networks were generated with WGCNA functions and visualized in Cytoscape v.3.7.2^2^.

### Selection of Sequences for HGT Inference and Phylogenetic Reconstruction

Homology searches, combined with multiple sequence alignment and distance calculations were used. As suggested by Li et al. (2018) for the assessment of HGT events in *R. irregulare*, an untargeted BLAST search is insufficient to assess similarity with sequences from distant taxonomical groups (i.e., bacteria and fungi). This is due to the fact that BLAST outputs are limited, and distant taxonomic groups may not be covered. According to this methodology, we first created three different subsets of the nr NCBI protein database (as of June 2019); one containing all bacterial sequences (Taxonomy ID: 2), one for the Dikarya (Taxonomy ID: 451864) and one for Mucoromycota (Taxonomy ID: 1913637). The KS and A domain of *G. margarita* KAF0502938.1 were then queried with BLASTp (Altschul et al., 1997) against the three databases separately. For both domains, five hundred BLAST hits were picked for Bacteria and Dikarya, while Mucoromycota BLAST hits were limited to few hundreds. To validate the protein dataset, each BLAST hit was fetched in its mRNA form with the Entrez Direct E-utilities^3^ to be analyzed with antiSMASH v.5 (Blin et al., 2019); this was needed to confirm that each protein in the datasets was potentially involved in the biosynthesis of secondary metabolites, as the antiSMASH v.5 pipeline needs a nucleotide input to work properly (Blin et al., 2019). Protein sequences which didn’t belong to a coding locus classified as PKS, NRPS, or NRPS-PKS were removed; for each remaining protein, only the regions that, according to BLASTp, had the highest similarity with the KS or A domains of KAF0502938.1 were kept in the datasets. The reduced KS and A sets produced this way for Bacteria, Dikarya and Mucoromycota were aligned separately with MAFFT v.7.132b (Katoh and Standley, 2013), and outliers and too identical sequences were removed with T-Coffee v.13.41 “+trim” command (Notredame et al., 2000), by setting a threshold of at least 25%, and at most 99% similarity. The resulting sets were merged, aligned and trimmed, so that each sequence in the final alignment had reciprocal similarities comprised between 27 and 99% for the KS domains dataset, and between 25 and 99% for the A domains dataset. The sequences of betaproteobacteria species, as the one from *Ca*Gg, did not survive the selection procedure, but were forcedly inserted in the analysis due to their importance for the hypothesis testing.

The alignments produced following the procedure described above were analyzed with prottest v.3.4.2 (Darriba et al., 2011) to select the best model of amino acid substitution (all distributions were tested). The best model according to Akaike’s information criterion (AIC) was LG+G+I for the KS dataset and the A dataset. The trees were produced with RAxML v.8.2.10 (Stamatakis, 2014) using the *autoMR* option of automatic “bootstopping” (Pattengale et al., 2010). Bayesian analyses were performed with MrBayes 3.2.6 (Ronquist et al., 2012) on the CIPRES portal (Miller et al., 2012). For each, two independent runs were performed with 500,000 generations. The number of chains was set to 8, the temperature parameter to 0.2, the sampling frequency was 10,000, and 25% of the samples were discarded as burnin. The output trees were midpoint rooted with figtree^4^ and manually edited.

In addition, we performed a constraint analysis to confirm the HGT origin of the KS domain of KAF0502938.1. The analysis is used for hypothesis testing and consists in the comparison of different tree topologies, with the aim of computing likelihood scores for each. In addition to the unconstrained tree, we generated two additional trees: in one tree, *Gigaspora* species were considered to be monophyletic with the Dikarya and *P. finnis*, i.e., it was assumed that the domain had a fungal origin. In addition, a tree was generated in which the *Gigaspora* species were constrained to be monophyletic with bacteria. All the trees were generated with RAxML v.8.2.10 using the LG+G+I model and the autoMR option, and the constraints were passed to the command line using the *-g* option. The three topologies were tested for significance in IQ-TREE v.1.6.12 (Minh et al., 2020).

### Molecular Analyses and Phylogenetic Reconstructions

Twenty-eight AMF spore isolates, belonging to seventeen different Gigasporaceae species, were investigated. For each isolate, 10–15 spores were surface sterilized as described by Lumini et al. (2007) and genomic DNA was extracted by using a CTAB-based method (Doyle and Doyle, 1990). To confirm the presence/absence of *Ca*Gg, a partial fragment of the 23S rRNA gene was amplified using Phusion^TM^ High-Fidelity Taq (Thermo Fisher Scientific, Waltham, MA, United States) with the primer pair GlomGIGf-GlomGIGr (Salvioli et al., 2008). The cycling conditions were the same used by Salvioli et al. (2008).

A partial fragment of the NRPS-PKS was amplified using Phusion^TM^ High Fidelity Taq with the new primers PKSf (5′-GCCTGTGCGTGCAAAAGCTACC-3′) and PKSr (5′-GGCCCATTGTCCAGTAGCA-3′). This primer pair targeted a region of about 730 bp from the hybrid NRPS-PKS KAF0502938.1, since this gene revealed the highest expression levels in all the fungal life stages (Venice et al., 2020), and has the best reciprocal BLAST hit (RIB14068.1) among the genes of the closely related *G. rosea*. The cycling conditions were: an initial step at 99°C for 3 min, 35 cycles of 98°C for 10 s, 60°C for 30 s, 72°C for 30 s and a final extension step at 72°C for 7 min. A partial fragment (∼700 bp) of the 28S rRNA gene was amplified using DreamTaq DNA polymerase (Thermo Fisher Scientific) with the primers LR1 (Van Tuinen et al., 1998) and 28G2 (Silva et al., 2006). The cycling conditions were: an initial step at 95°C for 5 min, 40 cycles of 94°C for 45 s, 56°C for 1 min, 72°C for 1 min and a final extension step at 72°C for 7 min.

Non-ribosomal peptide synthases-polyketide synthases amplicons were cloned using TOPO-TA cloning kit (Thermo Fisher Scientific). Clones were sequenced on an ABI 3730 capillary sequencer using BigDye v. 3.1 sequencing chemistry (Applied Biosystems, Foster City, CA, United States).

Sequences were assembled and curated in Geneious v. 8.1.7 (Kearse et al., 2012) and used as queries to conduct BLAST searches on GenBank (Benson et al., 2008). Sequences were then aligned with MAFFT (Katoh and Standley, 2013), Prior to phylogenetic reconstruction, best-fit nucleotide substitution model was estimated with jModelTest v.2.1.10 (Darriba et al., 2012). Phylogenetic reconstructions were carried out with RAxML v.8.2.10 (Stamatakis, 2014) and MrBayes v.3.2.7 (Ronquist et al., 2012). ML analyses were conducted with the *autoMR* option of automatic “bootstopping” (Pattengale et al., 2010) under GTRCAT (NRPS-PKS) and GTRGAMMA (28S rRNA gene) nucleotide substitution models. Markov chain Monte Carlo was run for 5 million generations under the TVM+G (NRPS-PKS) and TIM3+G (28SU rRNA gene) nucleotide substitution models.

## Data Availability Statement

Publicly available datasets were analyzed in this study. This data can be found here: *Gigaspora margarita* assembly: https://www. ncbi.nlm.nih.gov/assembly/GCA_009809945.1; *Gigaspora margarita* RNA-seq libraries: https://www.ncbi.nlm.nih.gov/bio sample/?term=(gigaspora%20margarita)%20AND%20biosample _sra[filter]%20AND%20public[filter]; *Gigaspora rosea* assembly: https://www.ncbi.nlm.nih.gov/assembly/GCA_003550325.1/; *Candidatus* Glomeribacter gigasporarum assembly: https://mage. genoscope.cns.fr/microscope/home/index.php.

## Author Contributions

FV, AD, AS, and PB designed the experiment. FV developed and performed all the bioinformatic analyses. AD, AS, and GS performed the amplicon-based experiment. AD performed the amplicon-based phylogenetic analysis. FV and PB wrote the manuscript. All authors contributed to the article and approved the submitted version.

## Conflict of Interest

The authors declare that the research was conducted in the absence of any commercial or financial relationships that could be construed as a potential conflict of interest.

## Footnotes

1 https://mycocosm.jgi.doe.gov/programs/fungi/1000fungalgenomes.jsf

2 https://cytoscape.org/index.html

3 https://www.ncbi.nlm.nih.gov/books/NBK179288/

4 http://tree.bio.ed.ac.uk/software/figtree/

## Supplementary Material

Supplementary Figure 1 Conservation of specific NRPS and their genomic surroundings in *Rhizoglomus* species. PFAM domains were predicted with BIG-SCAPE, also highlighting the presence in the conserved region of sexuality-related HMG-box protein.

Supplementary Figure 2 Prediction of putative BGC in *G. margarita* through co-expression analysis. Panels **(A,B)** represent the top 15 and 40 co-regulated genes, respectively, extracted from larger clusters of co-expressed genes calculated out of 24 RNA-seq libraries from different fungal life stages. Both networks contain genes with a calculated co-regulation coefficient >0.6 in a range from 0 (no correlation) to 1 (full correlation). Co-regulation is shown as interconnections between nodes (genes), revealing that each gene is interconnected with all the others in the networks. **(A)** contains two putatively co-regulated PKS genes, while **(B)** contains a hybrid NRPS-PKS. Both networks contain other genes that may be associated with secondary metabolites biosynthesis and transport, such as P450 monooxygenases and general substrate transporters. Gene annotations were retrieved from Venice et al. (2020).

Supplementary Figure 3 ML tree based on the A domain of KAF0502938.1, a hybrid NRPS-PKS of *G. margarita.* The sequence of *G. margarita*, as well as that of its endobacterium, *Ca*Gg, are highlighted. Differently from the KS domain (Figure 3), a clustering of *Gigaspora* sequences in a bacterial clade is not observed in the A tree. The sequence of the *Ca*Gg endobacterium clusters into a sub-clade which is well separated from the *Gigaspora* sequences, but which contains sequences from *Myxococcus*. Bootstrap support values are shown on the tree nodes. Thick lines indicate Bayesian posterior probability ≥95%.

Supplementary Figure 4 Phylogenetic reconstruction of LSU rRNA sequences from Gigasporales isolates. Isolates which were analyzed for the presence on NRPS-PKS in the present study are shown in bold. The tree shows the topology obtained with the Bayesian method; branches with Bayesian posterior probabilities ≥0.95 are thickened and ML bootstrap support values ≥70 are shown. The tree was rooted by *Pacispora scintillans*.

Supplementary Table 1 List of the RNA-seq libraries used for the co-expression analysis, and the related publications/projects.

Supplementary Table 2 List of BLAST homologs of *G. margarita* NRPS-PKS, KAF0502938.1, sorted by *E*-value. Except for hits from the closest sequenced relative, *G. rosea*, the list is dominated by bacterial homologs.

Supplementary File 1 List of topologies obtained for the KS domain of KAF0502938.1 by using different constraint analyses in RAxML. To confirm the bacterial signature of the domain, the unconstrained **(A)** topology was compared with other constrained topologies where *Gigaspora* species and bacteria **(B)** and all fungi **(C)** were considered to be monophyletic. For each tree, probabilities were calculated with the one-sided Kishino–Hasegawa test (p-KH; Kishino and Hasegawa, 1989), the Shimodaira–Hasegawa test (p-SH; Shimodaira and Hasegawa, 1999), and the Approximately Unbiased test (p-AU; Shimodaira, 2002). Bootstrap proportions according to the RELL method (bp-RELL; Kishino et al., 1990) and Expected Likelihood Weights (c-ELW; Strimmer and Rambaut, 2002) were also calculated. Plus, and minus signs indicate acceptance or rejection by each test. The topology assuming monophyly between *Gigaspora* and other fungi was rejected by all test, while the unconstrained topology and the one assuming monophyly between *Gigaspora* and bacterial sequences were comparable, as also observed for their log-likelihood scores. The unconstrained topology had the best log-likelihood, but constraining *Gigaspora* and bacterial sequences together only produced a limited shift, as also indicated by the delta log-likelihood value (deltaL).
