# Supplementary figures and images for: The Mosaic Architecture of NRPS-PKS in the Arbuscular Mycorrhizal Fungus Gigaspora margarita Shows a Domain With Bacterial Signature
Source: Front Microbiol. 2020 Nov 26;11:581313. doi: 10.3389/fmicb.2020.581313 (PMC7732545; doi:10.3389/fmicb.2020.581313)

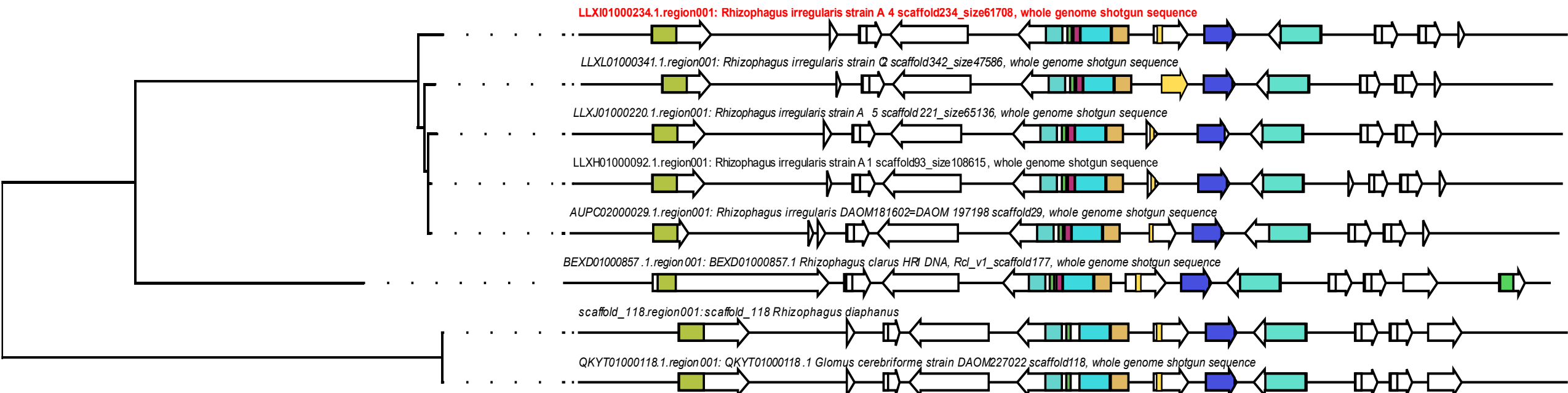

Supplement: Supplementary Figure 1 — Conservation of specific NRPS and their genomic surroundings in Rhizoglomus species. PFAM domains were predicted with BIG-SCAPE, also highlighting the presence in the conserved region of sexuality-related HMG-box protein. [file Data_Sheet_1.PDF]

A

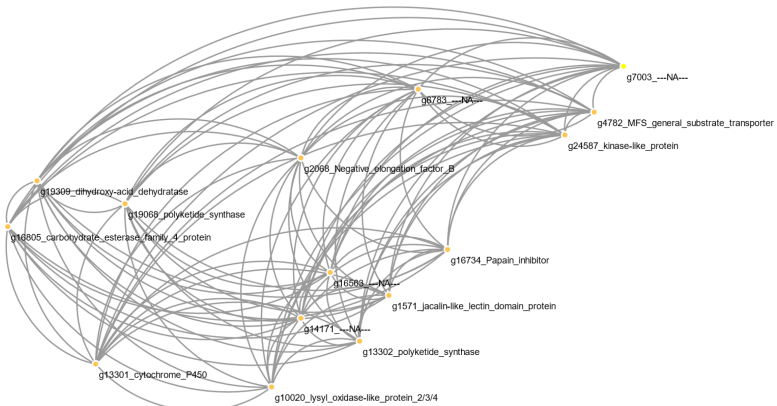

B

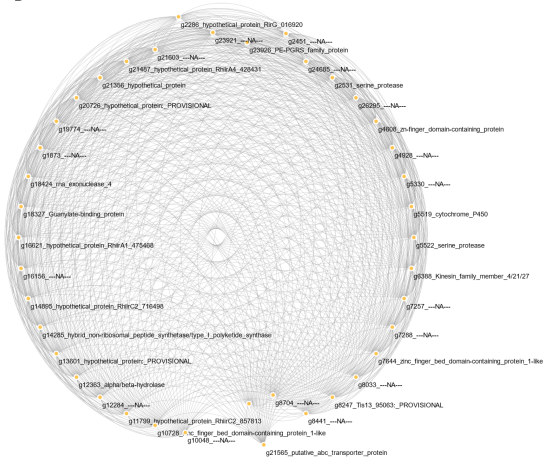

Supplement: Supplementary Figure 2 — Prediction of putative BGC in G. margarita through co-expression analysis. Panels (A,B) represent the top 15 and 40 co-regulated genes, respectively, extracted from larger clusters of co-expressed genes calculated out of 24 RNA-seq libraries from different fungal life stages. Both networks contain genes with a calculated co-regulation coefficient >0.6 in a range from 0 (no correlation) to 1 (full correlation). Co-regulation is shown as interconnections between nodes (genes), revealing that each gene is interconnected with all the others in the networks. (A) contains two putatively co-regulated PKS genes, while (B) contains a hybrid NRPS-PKS. Both networks contain other genes that may be associated with secondary metabolites biosynthesis and transport, such as P450 monooxygenases and general substrate transporters. Gene annotations were retrieved from Venice et al. (2020). [file Data_Sheet_2.PDF]

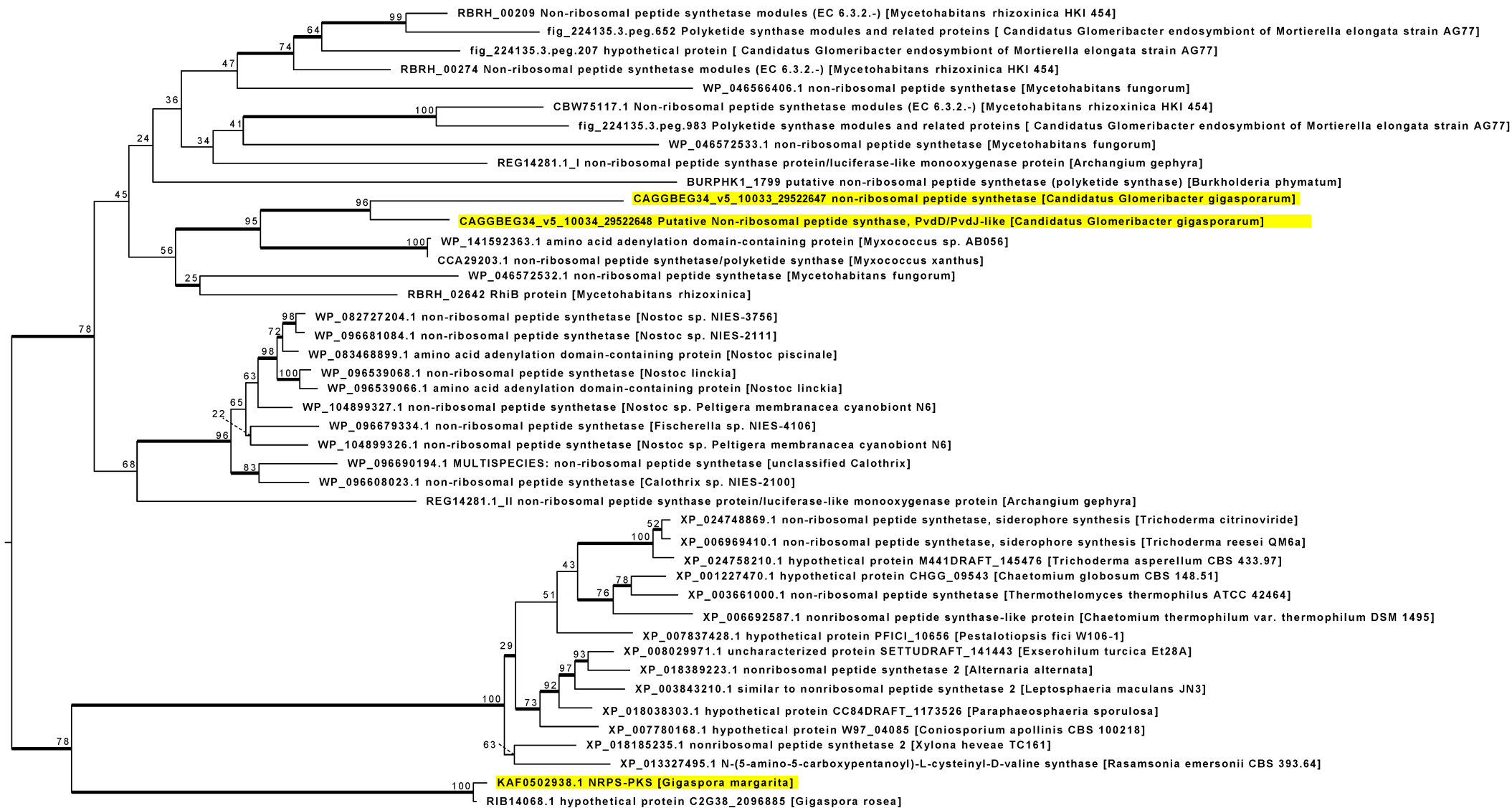

0.5

Supplement: Supplementary Figure 3 — ML tree based on the A domain of KAF0502938.1, a hybrid NRPS-PKS of G. margarita. The sequence of G. margarita, as well as that of its endobacterium, CaGg, are highlighted. Differently from the KS domain (Figure 3), a clustering of Gigaspora sequences in a bacterial clade is not observed in the A tree. The sequence of the CaGg endobacterium clusters into a sub-clade which is well separated from the Gigaspora sequences, but which contains sequences from Myxococcus. Bootstrap support values are shown on the tree nodes. Thick lines indicate Bayesian posterior probability ≥95%. [file Data_Sheet_3.PDF]

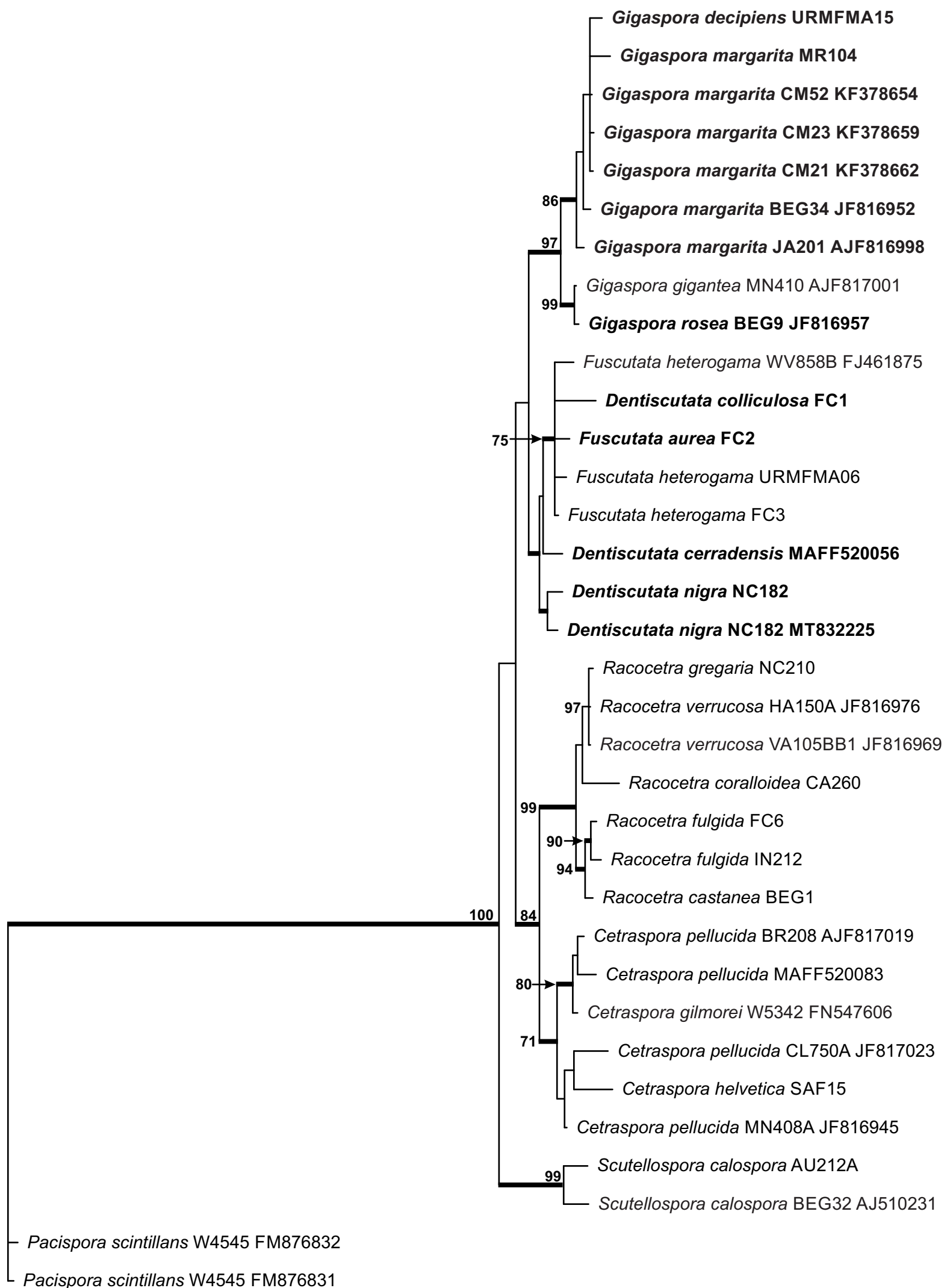

Supplement: Supplementary Figure 4 — Phylogenetic reconstruction of LSU rRNA sequences from Gigasporales isolates. Isolates which were analyzed for the presence on NRPS-PKS in the present study are shown in bold. The tree shows the topology obtained with the Bayesian method; branches with Bayesian posterior probabilities ≥0.95 are thickened and ML bootstrap support values ≥70 are shown. The tree was rooted by Pacispora scintillans. [file Data_Sheet_4.PDF]
