# Supplementary material for: The Mosaic Architecture of NRPS-PKS in the Arbuscular Mycorrhizal Fungus Gigaspora margarita Shows a Domain With Bacterial Signature
Source: Front Microbiol. 2020 Nov 26;11:581313. doi: 10.3389/fmicb.2020.581313 (PMC7732545; doi:10.3389/fmicb.2020.581313)
Supplement: Supplementary File 1 — List of topologies obtained for the KS domain of KAF0502938.1 by using different constraint analyses in RAxML. To confirm the bacterial signature of the domain, the unconstrained (A) topology was compared with other constrained topologies where Gigaspora species and bacteria (B) and all fungi (C) were considered to be monophyletic. For each tree, probabilities were calculated with the one-sided Kishino–Hasegawa test (p-KH; Kishino and Hasegawa, 1989), the Shimodaira–Hasegawa test (p-SH; Shimodaira and Hasegawa, 1999), and the Approximately Unbiased test (p-AU; Shimodaira, 2002). Bootstrap proportions according to the RELL method (bp-RELL; Kishino et al., 1990) and Expected Likelihood Weights (c-ELW; Strimmer and Rambaut, 2002) were also calculated. Plus, and minus signs indicate acceptance or rejection by each test. The topology assuming monophyly between Gigaspora and other fungi was rejected by all test, while the unconstrained topology and the one assuming monophyly between Gigaspora and bacterial sequences were comparable, as also observed for their log-likelihood scores. The unconstrained topology had the best log-likelihood, but constraining Gigaspora and bacterial sequences together only produced a limited shift, as also indicated by the delta log-likelihood value (deltaL). [file Presentation_1.PPT]

## Slide 1
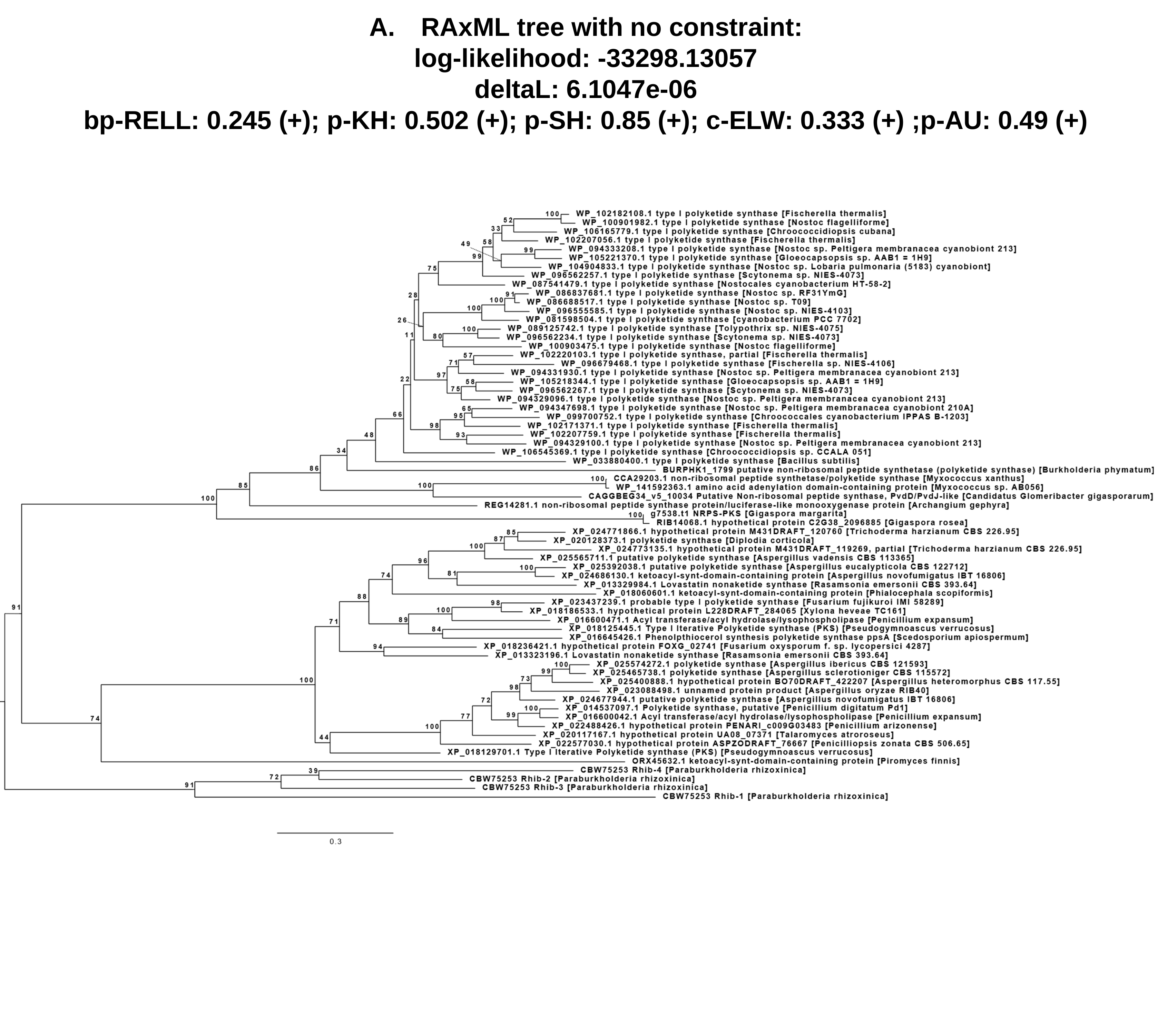

RAxML tree with no constraint:
log-likelihood: -33298.13057
deltaL: 6.1047e-06
bp-RELL: 0.245 (+); p-KH: 0.502 (+); p-SH: 0.85 (+); c-ELW: 0.333 (+) ;p-AU: 0.49 (+)

## Slide 2
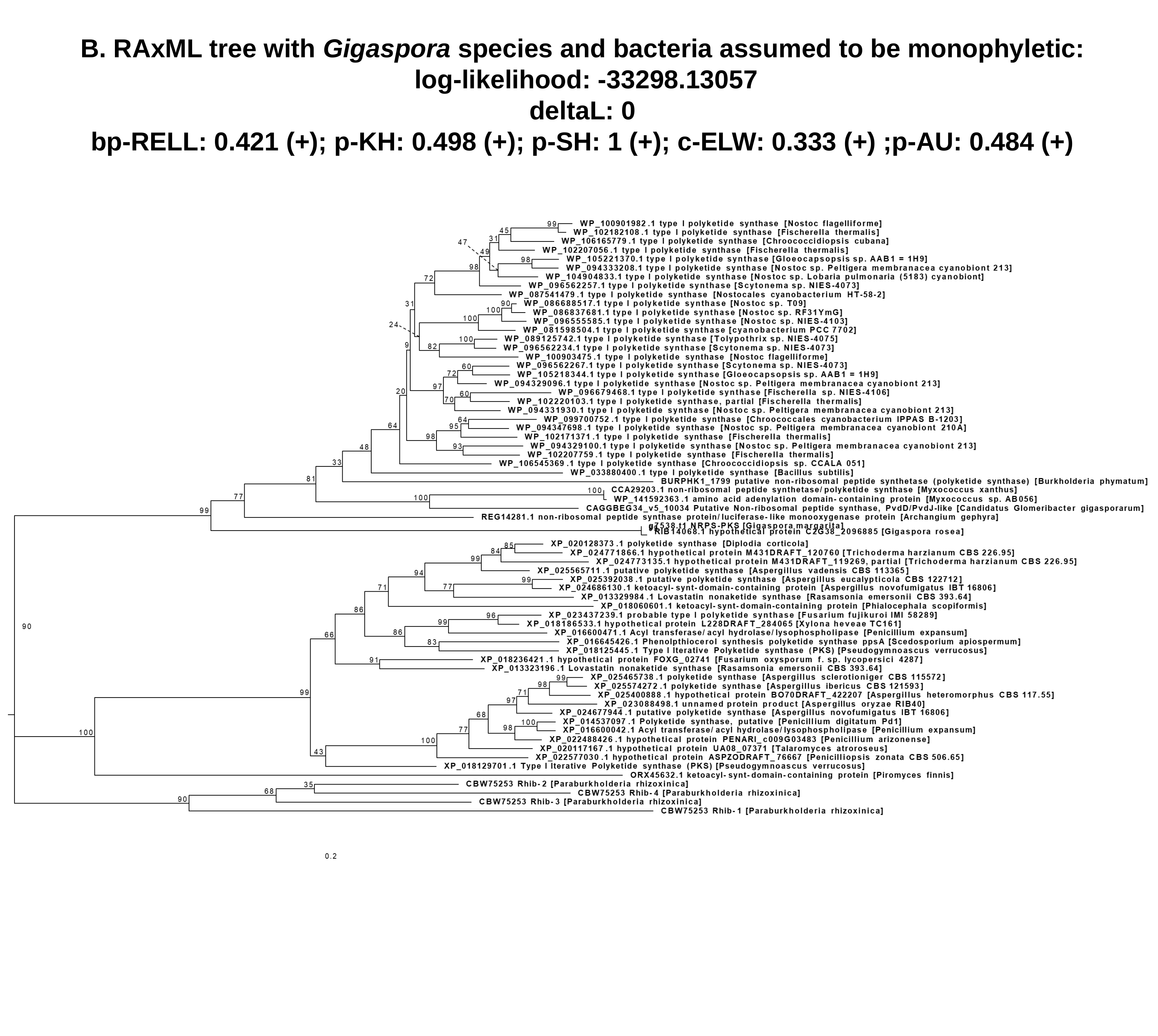

B. RAxML tree with Gigaspora species and bacteria assumed to be monophyletic:
 log-likelihood: -33298.13057
deltaL: 0
bp-RELL: 0.421 (+); p-KH: 0.498 (+); p-SH: 1 (+); c-ELW: 0.333 (+) ;p-AU: 0.484 (+)

## Slide 3
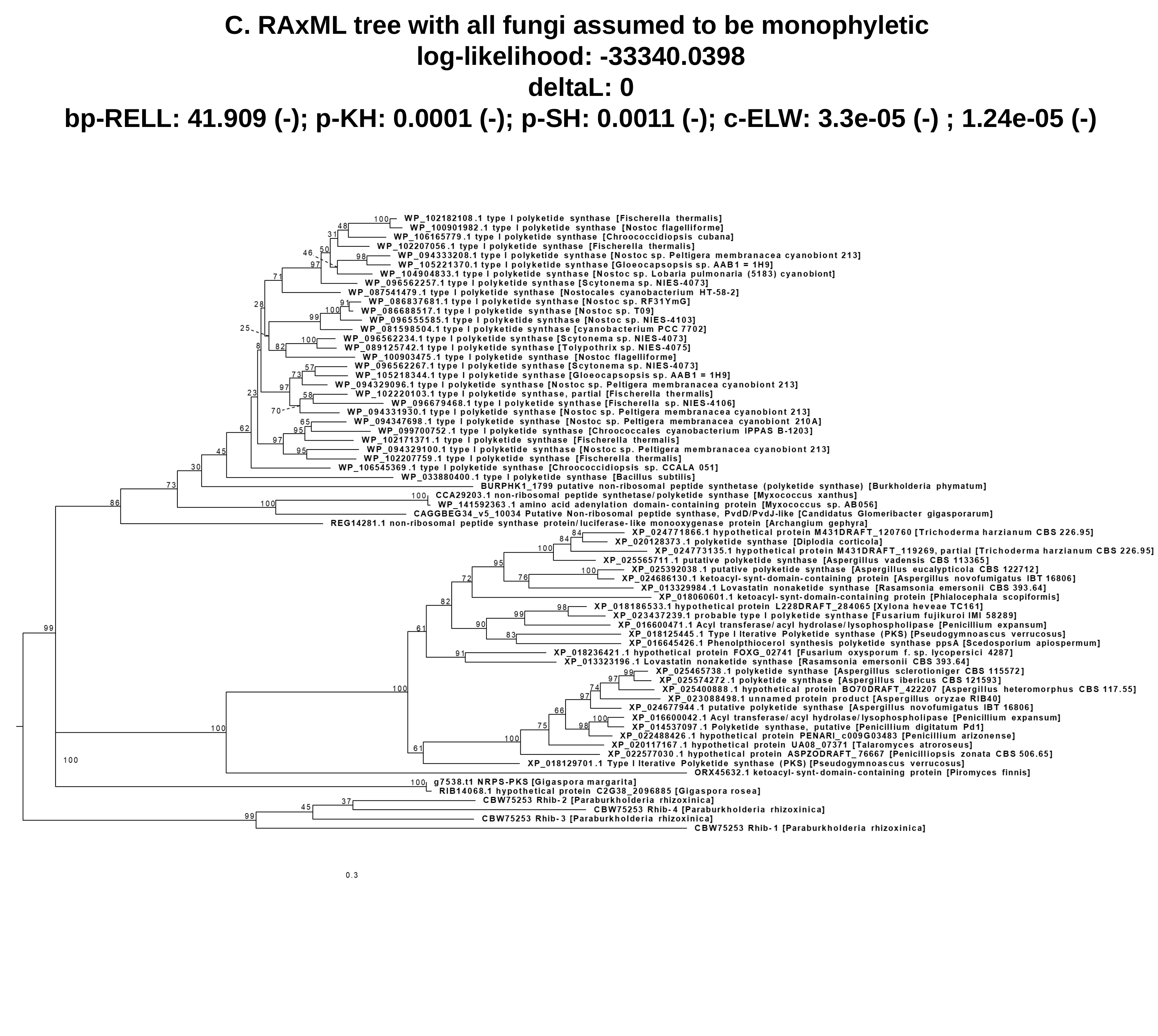

C. RAxML tree with all fungi assumed to be monophyletic
log-likelihood: -33340.0398
deltaL: 0
bp-RELL: 41.909 (-); p-KH: 0.0001 (-); p-SH: 0.0011 (-); c-ELW: 3.3e-05 (-) ; 1.24e-05 (-)
